# Modelling practices, data provisioning, sharing and dissemination needs for pandemic decision-making: a European survey-based modellers’ perspective, 2020 to 2022 

**DOI:** 10.2807/1560-7917.ES.2025.30.42.2500216

**Published:** 2025-10-23

**Authors:** Esther van Kleef, Wim Van Bortel, Elena Arsevska, Luca Busani, Simon Dellicour, Laura Di Domenico, Marius Gilbert, Sabine L van Elsland, Moritz UG Kraemer, Shengjie Lai, Philippe Lemey, Stefano Merler, Zoran Milosavljevic, Annapaola Rizzoli, Danijela Simic, Andrew J Tatem, Maguelonne Teisseire, William Wint, Vittoria Colizza, Chiara Poletto

**Affiliations:** 1 Centre for Tropical Medicine & Global Health, Nuffield Department of Medicine, University of Oxford, Oxford, United Kingdom; 2 Julius Center for Health Sciences and Primary Care, Utrecht University, Utrecht, the Netherlands; 3 Institute of Tropical Medicine, Antwerp, Belgium; 4 French Agricultural Research for Development (CIRAD), Unit for Animals, Health, Territories, Risks and Ecosystems (UMR ASTRE), French National Institute for Agricultural Research (INRAE), Montpellier, France; 5 Istituto Superiore di Sanità, Rome, Italy; 6 Spatial Epidemiology Lab, Université Libre de Bruxelles, Brussels, Belgium; 7 Department of Microbiology, Immunology and Transplantation, Rega Institute, KU Leuven, Leuven, Belgium; 8 Interuniversity Institute of Bioinformatics in Brussels, Université Libre de Bruxelles, Vrije Universiteit Brussel, Brussels, Belgium; 9 Institute of Social and Preventive Medicine, University of Bern, Bern, Switzerland; 10 MRC Centre for Global Infectious Disease Analysis, School of Public Health, Imperial College London, London, United Kingdom; 11 Department of Biology, Pandemic Sciences Institute, Oxford University, Oxford, United Kingdom; 12 WorldPop, School of Geography and Environmental Science, University of Southampton, Southampton, United Kingdom; 13 Department of Microbiology, Immunology and Transplantation, Rega Institute, KU Leuven, Leuven, Belgium; 14 Centre for Health Emergencies, Fondazione Bruno Kessler’s, Trento, Italy; 15 Institute of Public Health of Serbia “Dr Milan Jovanovic Batut”, Belgrade, Serbia; 16 Fondazione Edmund Mach, Research and Innovation Centre, San Michele all’Adige, Trento, Italy; 17 TETIS, INRAE, Montpellier, France; 18 Environmental Research Group Oxford Limited (ERGO), ℅ Department of Biology, Mansfield Road, Oxford, United Kingdom; 19 Sorbonne Université, INSERM, Pierre Louis Institute of Epidemiology and Public Health, Paris, France; 20 Department of Molecular Medicine, University of Padova, Padova, Italy

**Keywords:** modelling, outbreak analytics, pandemic decision-making, enhanced infectious disease surveillance, science-policy interface

## Abstract

**BACKGROUND:**

Advanced outbreak analytics were instrumental in informing governmental decision-making during the COVID-19 pandemic. However, systematic evaluations of how modelling practices, data use and science–policy interactions evolved during this and previous emergencies remain scarce.

**AIM:**

This study assessed the evolution of modelling practices, data usage, gaps, and engagement between modellers and decision-makers to inform future global epidemic intelligence.

**METHODS:**

We conducted a two-stage semiquantitative survey among modellers in a large European epidemic intelligence consortium. Responses were analysed descriptively across early, mid- and late-pandemic phases. We used policy citations in Overton to assess policy impact.

**RESULTS:**

Our sample included 66 modelling contributions from 11 institutions in four European countries. COVID-19 modelling initially prioritised understanding epidemic dynamics; evaluating non-pharmaceutical interventions and vaccination impacts later became equally important. Traditional surveillance data (e.g. case line lists) were widely available in near-real time. Conversely, real-time non-traditional data (notably social contact and behavioural surveys) and serological data were frequently reported as lacking. Gaps included poor stratification and incomplete geographical coverage. Frequent bidirectional engagement with decision-makers shaped modelling scope and recommendations. However, fewer than half of the studies shared open-access code.

**CONCLUSIONS:**

We highlight the evolving use and needs of modelling during public health crises. Persistent gaps in the availability of non-traditional data underscore the need to rethink sustainable data collection and sharing practices, including from for-profit providers. Future preparedness should focus on strengthening collaborative platforms, research consortia and modelling networks to foster data and code sharing and effective collaboration between academia, decision-makers and data providers.

**Key public health message**

**What did you want to address in this study and why?**

We wanted to know how COVID-19 modelling was used across Europe to support public health decisions. We evaluated changes in modelling practices, data access and collaboration with policymakers. To our knowledge, this is the first systematic and semiquantitative assessment of these elements during the pandemic, offering insights for better crisis response in the future.

**What have we learnt from this study?**

Modelling priorities shifted throughout the pandemic, from understanding the virus in the early stages to evaluating interventions such as vaccines later on. While timely case numbers were widely available, (real-time) behavioural, mobility and immunity data and sufficient population details were often missing. Collaboration between scientists and decision-makers evolved from informal network exchanges to formal advisory roles.

**What are the implications of your findings for public health?**

There is a need for rethinking the sustainability of existing and recently emerging collaborative platforms and advisory boards, including research consortia and modelling networks. This can help foster standardised data collection, sharing and coordination during pandemics, particularly for data that move beyond counting cases and come from diverse (including private) providers, so to act faster in future health emergencies.

## Introduction

The COVID-19 pandemic represented one of the most notable global health emergencies in recent years [1]. While the immediate urgency around COVID-19 has receded, the widespread circulation of Mpox clades I and II, alongside the risk from avian influenza H5N1, underscores the continuous occurrence of viral emergence events and the importance of investment in preparedness for new pandemic threats [2].

During the COVID-19 pandemic, governments and policymakers implemented detection, monitoring and intervention strategies in the face of an uncertain and rapidly evolving epidemiological landscape [3]. As such, COVID-19 exposed vulnerabilities in public health systems worldwide. To support decision-making, mathematical and computational approaches, commonly referred to as outbreak analytics, played a critical role [4-6]. This was accompanied by an unprecedented effort in the collection and sharing of epidemiological data such as case reporting, viral genome sequencing and serological data, and also non-traditional surveillance data, e.g. mobility flows reconstructed from mobile phones, geolocation, data on air travel, surveys on social mixing, and sentiment data from social media [7].

Reflections and commentaries by scientists, alongside high-level multilateral and expert reviews, have provided insights into lessons learned from modelling [7-9]. The discourse has focused on identifying modelling and data needs at different outbreak stages [6,7,10-20], developing efficient and flexible data collection frameworks that can rapidly scale up when necessary [21,22], improving communication and collaboration between modellers and public health authorities [8,23-27], and a rethinking of rewarding structures and institutional support for science policy activities [28].

Here, we contribute to these reflections with an objective and systematic reconstruction of the outbreak analytics activities conducted throughout the COVID-19 pandemic by MOOD (MOnitoring Outbreaks for Disease Surveillance in a data science context), multi-partner, multi-country epidemic intelligence consortium (mood-h2020.eu), spanning 25 partners. We identified how outbreak analytics’ scope, methodologies and input data evolved throughout the pandemic. We examined data sources, data limitations and missing data. We then reviewed the nature of interactions between scientists and decision-makers and the policy impact of modelling. Hence, this study aimed to provide insights into how quantitative scientific advice evolved, the extent to which it was implemented in policy, and the gaps in perceived needs between scientists and policymakers.

## Methods

### Study participants

The MOOD project is a Horizon2020 European Union (EU)-funded project (start date: January 2020, end date: December 2024), bringing together partners from academic, research, public health and animal health institutions. The project aimed to develop innovative tools and services for the early detection, assessment, and monitoring of current and potential infectious disease threats in the EU and European Economic Area (EEA) using a participatory approach [29]. Modelling teams within MOOD were located in four countries, i.e. France, Belgium, Italy and the United Kingdom (UK), with different socio-ecological (western, southern/Mediterranean and northern Europe) and epidemiological (organisation and structure of health services, state of health infrastructures, complexity of surveillance systems) contexts. At the same time, partners (and collaborations on the modelling work within MOOD) spanned a wider number of countries, including Italy, Serbia and Switzerland. Hence, partners of the MOOD consortium and the application of their work reflect important variation across the European region.

### Survey

Following a two-stage approach, we developed a semiquantitative survey for MOOD partners. In April 2022, we conducted a scoping survey with open-ended questions. We inventoried all modelling studies on the COVID-19 pandemic concluded until the survey date, here defined as encompassing computational (e.g. machine learning and phylogenetic approaches), statistical (e.g. spatial-temporal models, regression techniques) and mechanistic modelling (e.g. compartmental and agent-based models) studies. Based on answers in the scoping survey, we designed a final semiquantitative survey using similar inclusion criteria and predominantly closed questions; the full survey can be viewed in the Supplement. The survey was distributed among MOOD partners in July 2023. All studies carried out before the survey date were eligible for inclusion. These were studies conducted in collaboration with or assigned by public health authorities (PHA) and/or decision-makers, as well as those communicated to PHA (either directly or indirectly, e.g. via scientific publication), or neither.

The final survey covered questions on: (i) objectives of the work, (ii) methodologies used, (iii) data most frequently used and missing, (iv) data availability and access, and reason for lack of access, and (v) means of collaboration between scientists and decision-makers. In addition, for each study, the survey inventoried the pandemic period to which the study was referring (year and month of beginning, and year and month of end), code-sharing practices, the geographical scope of the work and the generalisability of the study to other diseases or geographical locations. Participants could select answers from a predefined list, with answers not mutually exclusive in many cases and the possibility to add free text to specify answers not included.

### Analyses

We analysed free text answers manually, and re-coded response categories where relevant. We classified studies according to whether their end date was aligned with the early, mid- or late pandemic phases, defined as (i) between January and June 2020 (dominating variant: severe acute respiratory syndrome coronavirus 2 (SARS-CoV-2) wild type), (ii) between July 2020 and June 2021 (SARS-CoV-2 Alpha wave), and (iii) from July 2021 (SARS-CoV-2 Delta and Omicron waves), respectively. Analyses involved descriptive statistics, cross-tabulation and stratification by pandemic period. We applied Fisher’s exact test to assess differences in modelling scope and data completeness by the degree and nature of science–policy interactions. As differences were tested for each modelling scope and data type separately, we applied a Bonferroni correction to adjust for multiple comparisons and control the type I error rate.

To measure policy impact, we used as a metric the number of policy documents that cited the respective modelling works identified in the Overton database. Overton is a global database of national and intergovernmental policy documents that allows tracking of policy citations, including citations to scientific evidence of interest. All Overton reports were extracted on 19 December 2024.

## Results

The final sample included 66 modelling contributions, with each survey response referring to a specific modelling study developed by the respondent’s team. These 66 studies were led by 11 institutions across four European countries (France, Belgium, Italy and the UK). The proportion of missing data was 3%. After manually resolving ambiguities, we assigned a pandemic phase to each of the contributions. Our sample included a close to equal number of studies for each pandemic phase, i.e. 24, 21 and 21 studies from the early, mid- and late pandemic phases, respectively. 

**Figure 1:**
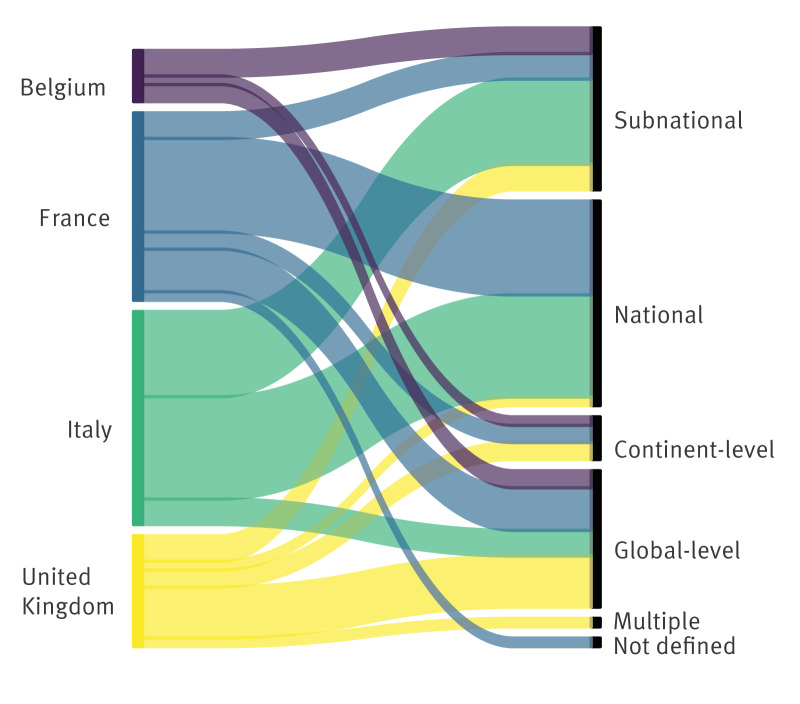
Geographical scope of the modellers’ survey on studies for COVID-19 pandemic decision-making, four European countries, March 2020–March 2022 (n = 66)

The geographical scope was defined for 65 of the 66 studies and notably included national (n = 24) and sub-national level (n = 19), followed by global (n = 16) and continent-level (n = 5) (Figure 1). The modelling work was considered applicable to other geographical locations for all works but two: in different locations with similar socio-demographic contexts for around one-third of studies (n = 21) and worldwide for two-thirds (n = 43). Finally, we considered most works applicable to other diseases (63/66). Around a third of the works (n = 21) leveraged openly available code. Conversely, fewer than half of the studies (n = 31) developed code that was made publicly available, with GitHub as a predominant outlet.

Alluvial diagram of the number of surveyed works by country vs geographical scope. ‘Multiple’ refers to more than one country, but not at global scale.

### Scope and analytical methods in the early and late COVID-19 pandemic phases (January 2020 to March 2022)

**Figure 2:**
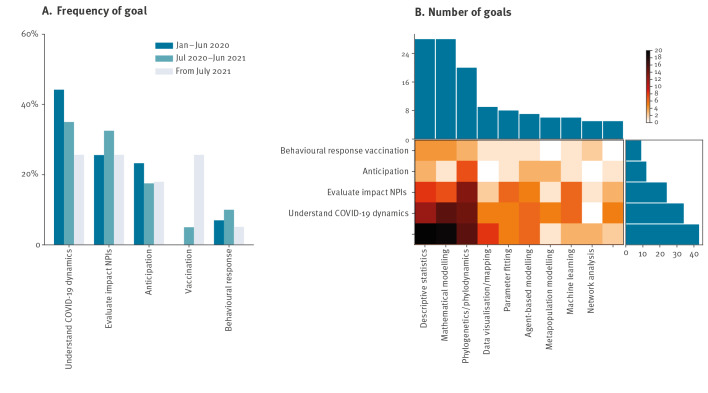
Scope and methodology of modelling works for COVID-19 pandemic decision-making, four European countries, January 2020–March 2022 (n = 66)

Overall, modelling work during the COVID-19 pandemic provided an understanding of SARS-CoV-2 dynamics (n = 43), that is, the estimation of transmission parameters (n = 14), quantification of the COVID-19 burden (n = 12), understanding of determinants of geographical spread (n = 7), estimation of the true number of cases/under-reporting (n = 8), and others (e.g. clinical aspects, emergence) (Figure 2A). Secondly, modelling assessed the impact of non-pharmaceutical interventions (NPIs, n = 34), including lockdown (n = 15), social distancing (n = 15) and travel restrictions (n = 14). Understanding the epidemic dynamics remained the most important focus, or among the most important, throughout the pandemic. In addition, the fraction of studies evaluating the impact of NPIs increased in the mid-pandemic phase, while the evaluation of vaccination became notable in the late pandemic phase (after June 2021).

NPI: on-pharmaceutical interventions.

Descriptive statistics and mathematical modelling were frequently employed (n = 28 for both methods), primarily to understand the COVID-19 transmission dynamics (Figure 2B). This was followed by mathematical modelling (n = 20), which was the preferred approach for studying the impact of vaccination and anticipating the course of the pandemic (Figure 2B).

### Data (re)sources

**Figure 3:**
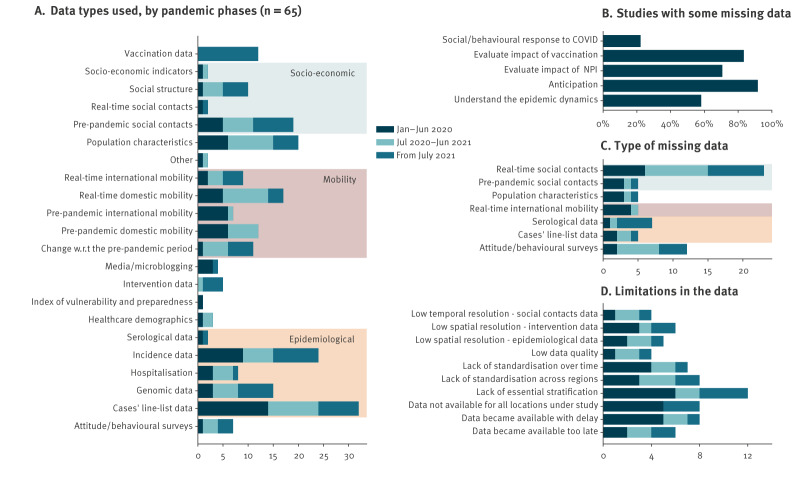
Data types used vs missing data in modelling studies for COVID-19 pandemic decision-making, four European countries, January 2020–March 2022 (n = 66)

The most widely adopted epidemiological data types included case line lists (n = 32), e.g. to estimate epidemiological transmission parameters such as generation time, basic-/net-reproduction number and incubation time (n = 9), and quantified the burden of COVID-19 (n = 8), followed by COVID-19 incidence data (n = 24) and genomic data (n = 15) (Figure 3A). Furthermore, mobility (n = 56; grouping all possible types of mobility data), socioeconomic data (n = 33; e.g. socioeconomic indicators, social contacts) and population characteristics (n = 20; including age, biological sex, ethnicity, comorbidities) were frequently sourced. Pre-pandemic and real-time mobility were used to inform models, notably during the early and mid-pandemic phases (Figure 3A). Conversely, vaccination, attitude/behavioural surveys, and social structure (household structure, school catchments, workplace size) were specifically sourced and used in the mid- and late phases.

NPI: non-pharmaceutical intervention; w.r.t.: with respect to.

The term Anticipation refers to prediction of the COVID-19 trajectory and/or future scenario analyses.

Overall, the data used became available because they were collected and shared in near real-time by PHA (n = 45), were gathered before the pandemic from freely and openly available sources (n = 25), or were collected specifically for and in the context of the modelling work of concern by study collaborators (n = 20). Interestingly, for-profit organisations were relevant data providers during the COVID-19 pandemic, sharing their data directly with modellers for a quarter of the studies (n = 16) or making the data openly available in the context of data-for-good initiatives (n = 16). This was the same for data shared through academic initiatives or initiatives of non-profit organisations (n = 15).

### Missing data and data access

For more than half of the modelling works, relevant data were missing (40/66). This increased slightly throughout the different phases of the pandemic, from 13 of 24 in the early phase to 14 of 21 in the late pandemic phase. Missing data primarily affected studies aimed at anticipating the course of the pandemic (22/24), followed by those assessing the impact of vaccination (10/12) or of NPIs (24/34) (Figure 3B). Particularly lacking were real-time social contact data (n = 23) and data on attitudes and behaviours (n = 12) (Figure 3C). The reported relative lack of these data remained similar throughout the pandemic.

The main reasons listed for missing data were that the data were never collected (n = 27), the process for obtaining them was too lengthy (n = 9), or the data were protected by privacy or ethical restrictions (n = 7). Where data were available, limitations applied in 39 studies, most notably related to a lack of essential stratification (e.g. incidence by age, sex and comorbidity, n = 12), delay in data availability (n = 8), lack of available data for all locations under study (n = 8), or lack of standardisation across regions (n = 8) (Figure 3D).

### Interaction with public health authorities

**Figure 4:**
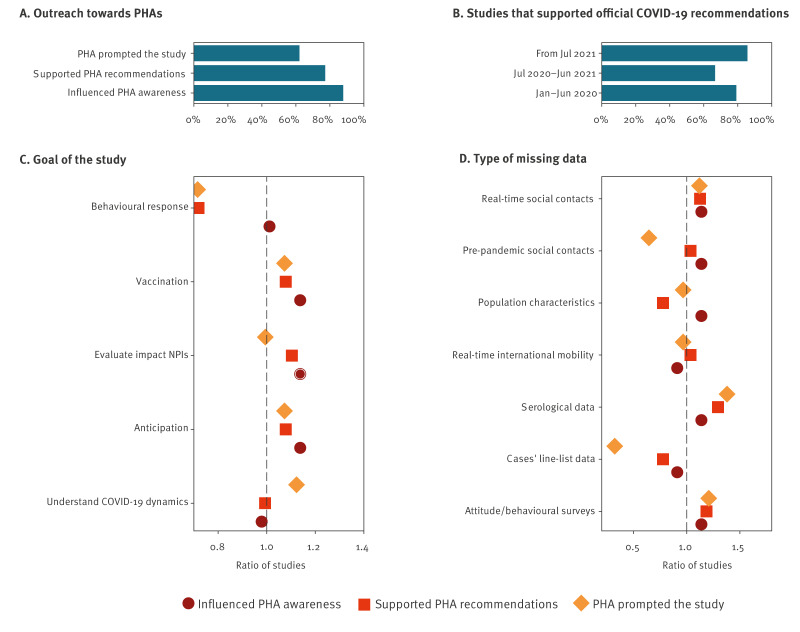
Interaction with public health authorities in modelling studies for pandemic decision-making, four European countries, January 2020–March 2022 (n = 66)

NPI: non-pharmaceutical intervention; PHA: public health authority.

For most modelling contributions (58/66), survey participants listed that their work supported the scientific understanding or situational awareness of decision-makers or PHA (Figure 4A). For a smaller but marked fraction, participants perceived that their work supported PHA official recommendations (51/66) and/or was directly prompted by a discussion with PHA (41/66). Direct interactions with PHAs occurred for 54 of the 66 studies and involved direct collaboration (45/54), discussions at internal meetings (4/54) or participation of modellers in advisory committees (5/54). Direct collaborations were noted particularly during the early pandemic phase (18/24, 14/21 13/21 reporting direct collaboration with PHA in the early, mid- and late pandemic phases respectively), while interactions through advisory committees occurred during the mid- and late pandemic phases (0/24, 3/21 and 2/21 reporting interaction through advisory committees in the early, mid- and late pandemic phases respectively).

In Panels C and D, the ratio refers to showing the ratio of the proportion of studies with a particular scope among each of three different possible interactions with PHA divided by the proportion of studies with that same objective across all studies (e.g. 73% of the studies prompted by PHA concerned understanding the epidemic dynamics, while this was 65% across all studies, which results in a ratio of 1.12). According to Fisher’s exact test, with p < 0.05 after correction for multiple testing, the only data point with statistical significance was the combination of the goal Anticipation with the outcome Influenced PHA awareness (marked with a white ring).

The number of studies supporting official recommendations was highest in the late pandemic phase, immediately followed by the early phase (Figure 4B). Among the 58 studies that supported situational awareness of PHA, the percentage of studies that evaluated the impact of NPIs (n = 34/58) was larger than their relative representation among all the modelling studies (i.e. including the studies that did not report an interaction with PHA (34/66), Fisher’s exact test p < 0.05, after correcting for multiple comparisons) (Figure 4C).

An analysis of the relative representation of missing data across studies according to their outreach towards PHAs (Figure 4D) illustrated that the lack of real-time social contacts, serological data and attitude/behavioural surveys was felt more frequently for all three kinds of outreach when compared with their relative reported lack across all studies. However, Fisher’s exact test with Bonferroni correction (p > 0.05) did not indicate substantial differences in missing data between studies with and without reported interactions with PHAs.

Our analysis of the public health impact of the modelling studies showed that 34 of the 66 studies were cited at least once in a policy document, resulting in a total of 121 citations in 103 unique policy documents from 17 countries, from the European Region and from international governmental organisations, in nine different languages. Studies of the early and mid-pandemic phases had more policy citations than studies of the late pandemic phase (average number of policy citations per study: 2.2, 2.0 and 1.3, respectively for early, mid- and late pandemic phases, respectively. An additional analysis of the policy relevance of the included studies is appended in Supplementary Figure S1. Studies aiming at anticipation of the pandemic trajectory and evaluating the impact of NPIs had, on average, more policy citations (2.8 and 2.9, respectively). Supplementary Figure S1 further summarises the number of policy citations of surveyed studies and average number of policy citations per study by goal of the study and pandemic period.

## Discussion

By evaluating COVID-19 outbreak analytics practices in four European countries and across different pandemic phases, we illustrated that evolving and complex public health needs drove the scope of analytical efforts. Similar to previous public health emergencies [30], early modelling focused on understanding epidemic dynamics such as transmission parameters and disease burden. Over time, attention shifted towards evaluating NPIs and later on to vaccination strategies, as UK-based modelling reported previously [5]. Nonetheless, understanding severe acute respiratory syndrome coronavirus 2 (SARS-CoV-2) transmission remained central throughout, as emerging viral variants and changes in affected population characteristics, such as waning and growing immunity, required ongoing reassessment of key epidemiological parameters.

Evolving outbreak phases shape data use and requirements [30,31], with the long-term nature of the COVID-19 pandemic revealing both advances and gaps in existing disease surveillance systems and data pipelines. We found that modellers’ data needs were met for ca 40% of the studies. Two-thirds of studies relied on surveillance data. Given the persistent need to reassess epidemiological parameters, case line lists, incidence rates and genetic data remained fundamental requirements during all pandemic phases, as also corroborated by Jit et al.’s reflections and experiences with COVID-19 pandemic modelling in western European countries [7]. Furthermore, fluctuating epidemic waves prolonged the need to (re-) assess optimal NPIs in a continuously changing epidemiological context. Among non-traditional data sources, mobility data were used in half of the studies and were earlier identified as critical by modellers from France, the Netherlands and the UK [7]. As PHA sought to balance health, social and economic costs, non-traditional data sources such as real-time mobility [32], social contact data [33] and behavioural surveys became increasingly needed to anticipate complex interactions between the virus’ epidemiology and the behavioural response to interventions in a context of epidemic fatigue and misinformation.

In some cases, real-time data collection and linkage were made possible by innovative coordination frameworks, built during the emergency and involving the integration of governmental bodies, academia and the private sector. Examples include the REACT programme to track the progress of England’s epidemic through home testing [34] and the COG-UK (https://www.cogconsortium.uk) and EMERGEN consortia for virus sequencing in the UK and France, respectively. Initiatives for collating, curating and standardising data from diverse sources, e.g. Global.health, ourworldindata.org and initiatives like the European Centre for Disease Prevention and Control’s scenario hub made data readily available for modelling, limiting barriers and delays in data acquisition and processing on surveillance data. We found that for-profit organisations were the main source of mobility data and the second largest source of data overall by either selling or sharing the data directly with modellers under non-disclosure agreements (IATA, mobile phone companies, Google and Facebook) or making coarsely aggregated data openly available (e.g. COVID-19 community mobility reports by Google, data not shown). Other relevant data on human behaviour were collected by research consortia funded by the European Commission, e.g. CoMix, to collect real-time social mixing data in over 20 countries [35].

Our study showed that despite these efforts, necessary data were often not available to modellers when needed, most notably real-time social contact and attitude/behavioural information, followed by serological data. In part this delay may be due to the time needed to secure funding and set up academic consortia (as these data were not routinely available before the pandemic). Moreover, according to survey respondents, data did not cover sufficient study locations and/or lacked essential stratification and standardisation. Kraemer et al. recently provided an overview of how advances and availability of artificial intelligence methods have the potential to more rapidly integrate disparate data sources, enabling rapid risk assessments and providing guidance for policymakers during health emergencies [36]. They are however dependent on the availability of robust data, which remains one of the main barriers for their deployment. Enhanced data collection campaigns should be made protocol-ready and scalable already before a future pandemic to increase geographical coverage, sustainable data collection and operational readiness. Similar to the First Few X cases or the UNITY study protocols [37], standardised data collection protocols for non-traditional surveillance data could be formulated through continuous collaboration between academic and public health sectors on the one hand and for-profit data providers on the other. 

Existing participatory platforms similar to Influenzanet.info could be leveraged to deploy and scale up surveys rapidly. The Social Study (thesocialstudy.be), set up by Belgian Universities in collaboration with federal and local governments, aims to follow a representative group of community members and their opinions on various topics by deploying surveys regularly in non-pandemic times. Such open-source data initiatives help further integrate behavioural and sentiment data in modelling frameworks, mitigate misinformation [38] and monitor risks [39].

In all four countries in our study, modelling substantially informed COVID-19 pandemic decision-making at PHA. In particular, there was a need to understand the impact of NPIs. Indeed, studies with this aim were more represented among the studies that supported PHAs’ scientific understanding and were also among the ones, together with pandemic anticipation studies, that obtained more policy citations. We furthermore found an evolving integration of modelling into decision-making. Advisory committees were increasingly established during the mid- and late pandemic phases, with their multi-disciplinarity growing to address growing complexities (e.g. the Belgian pandemic management committee, the French Covid-19 Scientific Advisory Board, and SPI-M-O in the UK), thus exchanges of requests and modelling frameworks used became more streamlined and transparent [7,24]. In the early phase of the COVID-19 pandemic, when epidemiological uncertainty was highest and rapid public health decisions were needed, informal collaborative networks across European countries – often built through pre-existing or earlier funded European Commission-funded consortia – facilitated the rapid exchange of epidemiological parameters, methods and emerging data.

Such pre-existing collaborations amplified the impact of formal structures, thus reinforcing the importance of sustained investment in cross-border modelling networks for pandemic preparedness. Building on this experience, national governments, including those in the EU/EEA, are investing in pandemic preparedness and resilience in the post-COVID era. They establish national and regional pandemic intelligence systems and networks of modellers and public health actors (e.g. European Centres of Disease Control’s respicast.ecdc.europa.eu and respicompass.ecdc.europa.eu, influcast.org, the World Health Organisation’s epi-parameter community, the Dutch Institute of Public Health and the Environment’s modelling platform for policymakers, and the Center for Forecasting and Outbreak Analytics at the United States Centers for Disease Control and Prevention). 

In a time where open source and open data are advocated, nearly half of the surveyed studies made code openly available. Delayed availability often stemmed from the need to prioritise rapid outputs for outbreak response over producing well-documented, usable code. Creating collaboration frameworks and increasing incentives or academic credits for code- and data-sharing could facilitate more and open data sharing. This in turn, would support the development of flexible analytical frameworks for different disease models during non-crisis periods [40], which can enhance reproducibility and cross-country comparability.

A limitation, but simultaneously a strength of our work, is that data were provided by modelling groups from four European countries. While our sample may not represent the full diversity of modelling practices and perspectives across the EU/EEA, the accompanying policy analysis shows that their work informed decision-making globally in a wide range of countries, supporting the broader relevance of their insights. Secondly, non-traditional economic and proxy- or environmental data sources were not listed among the data sources used and missing, while these have reportedly been used to inform COVID-19 modelling work. Similarly, in terms of modelling methodology, our study did not document the usage of ensemble modelling and comparative analyses. This may relate to our standardised answers format, which lacks some of these mentions. However, as we used a scoping survey to define our answer categories, we believe our responses are largely representative. Finally, as highlighted by others [41], quantification and communication of uncertainty of model plays a key role in ensuring appropriate policy translation and uptake. However, as we aimed to document the evolving scope, purpose and policy relevance of modelling contributions during the COVID-19 pandemic, with a focus on the interface between data availability, modelling and public health response, we believed this was outside of our study’s scope.

## Conclusion

Moving forward, pandemic preparedness requires sustainable and standardised data systems that go beyond case counts to include risk monitoring, demographic and geographical stratification, and ready-to-deploy protocols. Frameworks for ethical, long-term access to data from both public and private providers, along with open code sharing, are essential to enhance comparability, transparency and reproducibility. Collaboration between scientists and decision-makers, which evolved during the pandemic from informal exchanges to formal advisory structures, should be maintained and strengthened through cross-border modelling networks and collaborative platforms. Our findings can support and inform the development of national and regional pandemic intelligence systems currently being established across Europe and globally, and help shape future policy, technical approaches and communication strategies. Our approach can be further expanded to other geographical areas and emerging disease threats. Linking our approach with WHO’s Preparedness and Resilience for Emerging Threats (PRET) initiative, priority disease models based on modes of transmission can be defined and used to map further enhanced surveillance data needs, guide flexible analytical framework development, map actors and build collaborative networks.

## Data Availability

The code to perform the analyses reported in the paper is available at https://github.com/chiara-poletto/Modelling-practices

## Supplementary Data

389000 Supplement
